# Cells of the human respiratory tract support the replication of pathogenic Old World orthohantavirus Puumala

**DOI:** 10.1186/s12985-021-01636-7

**Published:** 2021-08-17

**Authors:** Stefan Hägele, Christian Nusshag, Alexander Müller, Alexandra Baumann, Martin Zeier, Ellen Krautkrämer

**Affiliations:** Department of Nephrology, University of Heidelberg, Im Neuenheimer Feld 162, 69120 Heidelberg, Germany

**Keywords:** Orthohantavirus, Lung, Endothelium, Epithelium, Puumala virus, Integrin, Receptor

## Abstract

**Background:**

Transmission of all known pathogenic orthohantaviruses (family *Hantaviridae*) usually occurs via inhalation of aerosols contaminated with viral particles derived from infected rodents and organ manifestation of infections is characterized by lung and kidney involvement. Orthohantaviruses found in Eurasia cause hemorrhagic fever with renal syndrome (HFRS) and New World orthohantaviruses cause hantavirus cardiopulmonary syndrome (HCPS). However, cases of infection with Old World orthohantaviruses with severe pulmonary manifestations have also been observed. Therefore, human airway cells may represent initial targets for orthohantavirus infection and may also play a role in the pathogenesis of infections with Eurasian orthohantaviruses.

**Methods:**

We analyzed the permissiveness of primary endothelial cells of the human pulmonary microvasculature and of primary human epithelial cells derived from bronchi, bronchioles and alveoli for Old World orthohantavirus Puumala virus (PUUV) in vitro. In addition, we examined the expression of orthohantaviral receptors in these cell types. To minimize donor-specific effects, cells from two different donors were tested for each cell type.

**Results:**

Productive infection with PUUV was observed for endothelial cells of the microvasculature and for the three tested epithelial cell types derived from different sites of the respiratory tract. Interestingly, infection and particle release were also detected in bronchial and bronchiolar epithelial cells although expression of the orthohantaviral receptor integrin β_3_ was not detectable in these cell types. In addition, replication kinetics and viral release demonstrate enormous donor-specific variations.

**Conclusions:**

The human respiratory epithelium is among the first targets of orthohantaviral infection and may contribute to virus replication, dissemination and pathogenesis of HFRS-causing orthohantaviruses. Differences in initial pulmonary infection due to donor-specific factors may play a role in the observed broad variance of severity and symptoms of orthohantavirus disease in patients. The absence of detectable levels of integrin α_V_β_3_ surface expression on bronchial and small airway epithelial cells indicates an alternate mode of orthohantaviral entry in these cells that is independent from integrin β_3_.

## Introduction

Infections with orthohantaviruses, formerly named hantaviruses, cause two diseases: hemorrhagic fever with renal syndrome (HFRS) by Old World orthohantaviruses found in Eurasia and hantavirus cardiopulmonary syndrome (HCPS) by New World orthohantaviruses of the American continents [1, 2]. HFRS is characterized by acute kidney injury, whereas the cardio-pulmonary involvement predominates in HCPS [3, 4]. However, the reasons for the virus-specific organ manifestations in both diseases despite transmission of all orthohantaviruses by inhalation are not known.

The clinical picture of infectious diseases depends on the tropism of the invading pathogen. Infection of target cells contributes to the clinical symptoms and is responsible for the dysfunction or even failure of the affected organ. Histopathological analysis of autopsy samples derived from HCPS patients infected with the New World orthohantavirus Sin Nombre (SNV) demonstrates that viral antigen was found in a number of organs such as lung, kidney, spleen, intestine, or lymph nodes [5]. The presence of viral antigen in different organs was also described for HFRS patients. In addition to infected cells in the kidney, viral antigen was also detected in the lung, intestine, in cells from bronchoalveolar lavage, in endothelial cells of the parotid gland, and RNA of Old World orthohantavirus Puumala virus (PUUV) was found in saliva [6–12]. Several cell culture studies in the African Green Monkey cell line Vero E6 and primary endothelial cells of the human umbilical vein (HUVEC) identified integrin β3 as entry receptor for pathogenic orthohantaviruses such as Sin Nombre virus, New York virus, Hantaan virus (HTNV), PUUV, Seoul virus, and Dobrava-Belgrade virus (DOBV); in addition, CD55 was described as co-receptor for entry of HTNV, PUUV, and DOBV [13–17]. Receptor expression and usage determines the tropism of viruses. However, the expression and the role of integrin β3 and CD55 for the entry in cells of orthohantaviral target organs have not been analyzed in detail so far.

Detection of orthohantaviral genomes and antigen in endothelial and epithelial cells of various tissues indicates a broad range of organ tropism and may explain the extrarenal manifestations observed in Old World orthohantavirus infection. HFRS-cases complicated by acute respiratory distress syndrome caused by HTNV are described in China and cases with severe respiratory symptoms are reported in the context of PUUV infections in Europe [9, 18–22]. The underlying mechanisms for the renal-pulmonary presentation of these infections caused by HTNV and PUUV are not completely understood. Direct virus-induced and immune response mediated effects as well as multiorgan dysfunction may contribute to the observed respiratory symptoms [3]. Tissue-specific damage is often caused by immune cell-mediated cytotoxicity that takes place at the site of viral infection to eliminate infected cells. As shown for New World orthohantaviruses, patients with PUUV infection also demonstrate pulmonary pathogenesis due to local cellular immune response in the airways [20, 23–26].

Orthohantaviruses are transmitted via inhalation of aerosols containing virions excreted from infected host animals. Therefore, cells of the respiratory tract may represent initial targets for infection by inhaled particles. However, replication of Old World orthohantaviruses in lung cells is not investigated in detail so far. To analyze if pulmonary cells may serve as primary entry site in human infections with Old World orthohantavirus PUUV, we examined receptor expression and infection of human primary cells derived from different parts of the respiratory tract.

## Material and methods

### Cells

Primary human pulmonary microvascular endothelial cells (HPMEC), human small airway epithelial cells (HSAEpC), and human bronchial epithelial cells (HBEpC) were obtained from PromoCell (Germany). Human pulmonary alveolar epithelial cells (HPAEpiC) were purchased from ScienCell (USA). The cells were maintained in Endothelial Cell Growth Medium MV2, Small Airway Epithelial Growth Medium, Airway Epithelial Growth Medium (all from PromoCell), and Alveolar Epithelial Cell Medium (ScienCell). Only cells from passages two to six were used. Cells derived from two different donors were analyzed for every cell type. Each individual is indicated by a three digit numeral identity code: HPMEC: donor #011 and #016; HBEpC: donor #359 and #404; HSAEpC: donor #210 und #306; HPAEpiC: donor #144 and #167. Cells were seeded at 10,000 cells/cm^2^. Vero E6 cells were maintained in Dulbecco’s modified Eagle’s medium (DMEM) supplemented with 10% fetal calf serum.

### Virus infection and quantification of infection

**Fig. 1:**
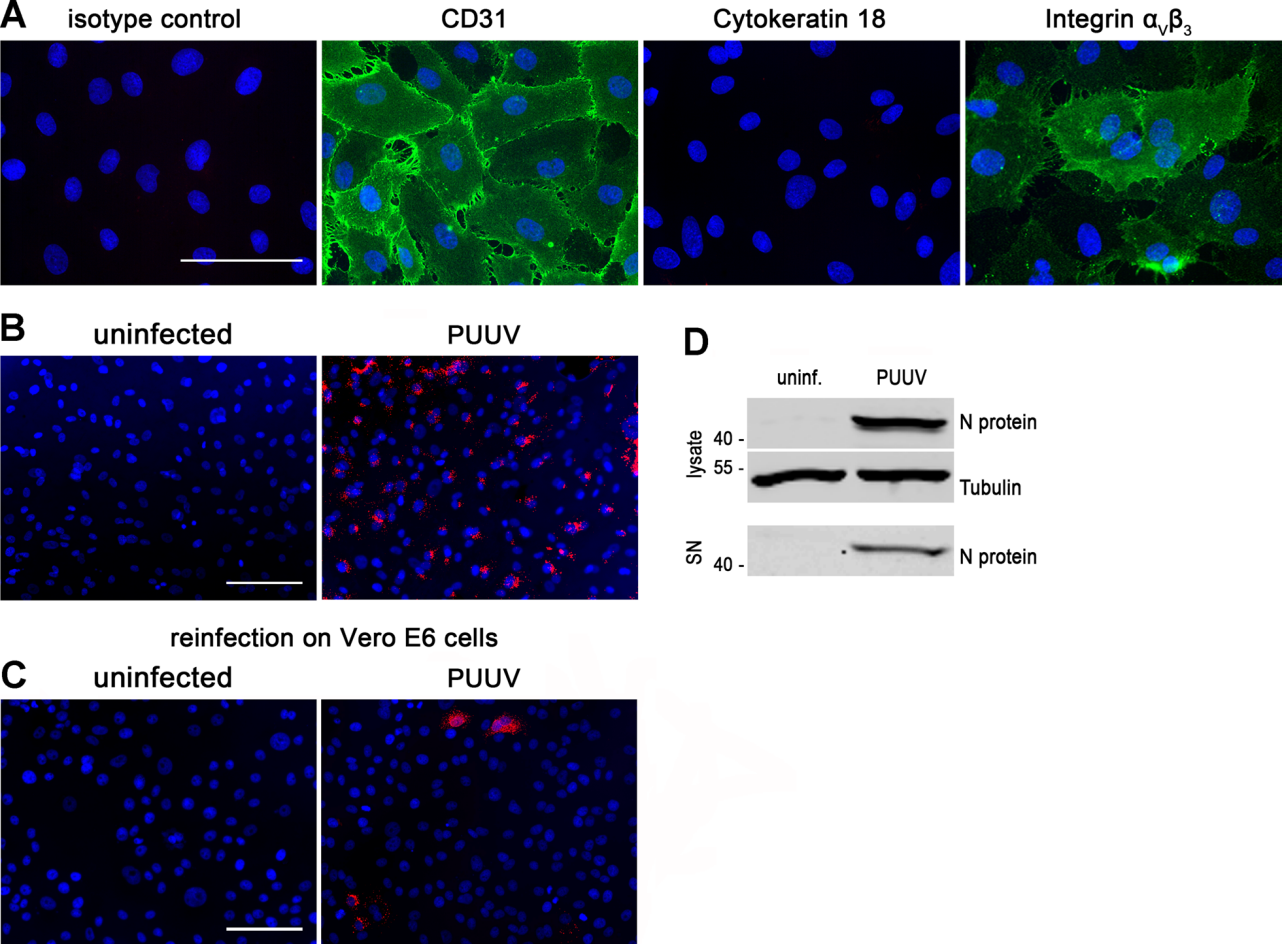
Infection of HPMECs with PUUV. **A** HPMECs were stained for CD31, cytokeratin 18 and integrin α_V_β_3_ (scale bar: 100 µm). **B** Cells were infected with PUUV and analyzed for expression of N protein by immunofluorescence on day 6 post infection (scale bar: 100 µm). Cellular lysates and cell-free supernatants (SN) were examined for N protein by Western blot analysis on day 6 post infection. **C** Vero E6 cells were incubated with cell-free supernatants derived from uninfected or infected HPMECs (6 dpi) and analyzed for N protein expression after 48 h post infection (scale bar: 100 µm)

**Fig. 2:**
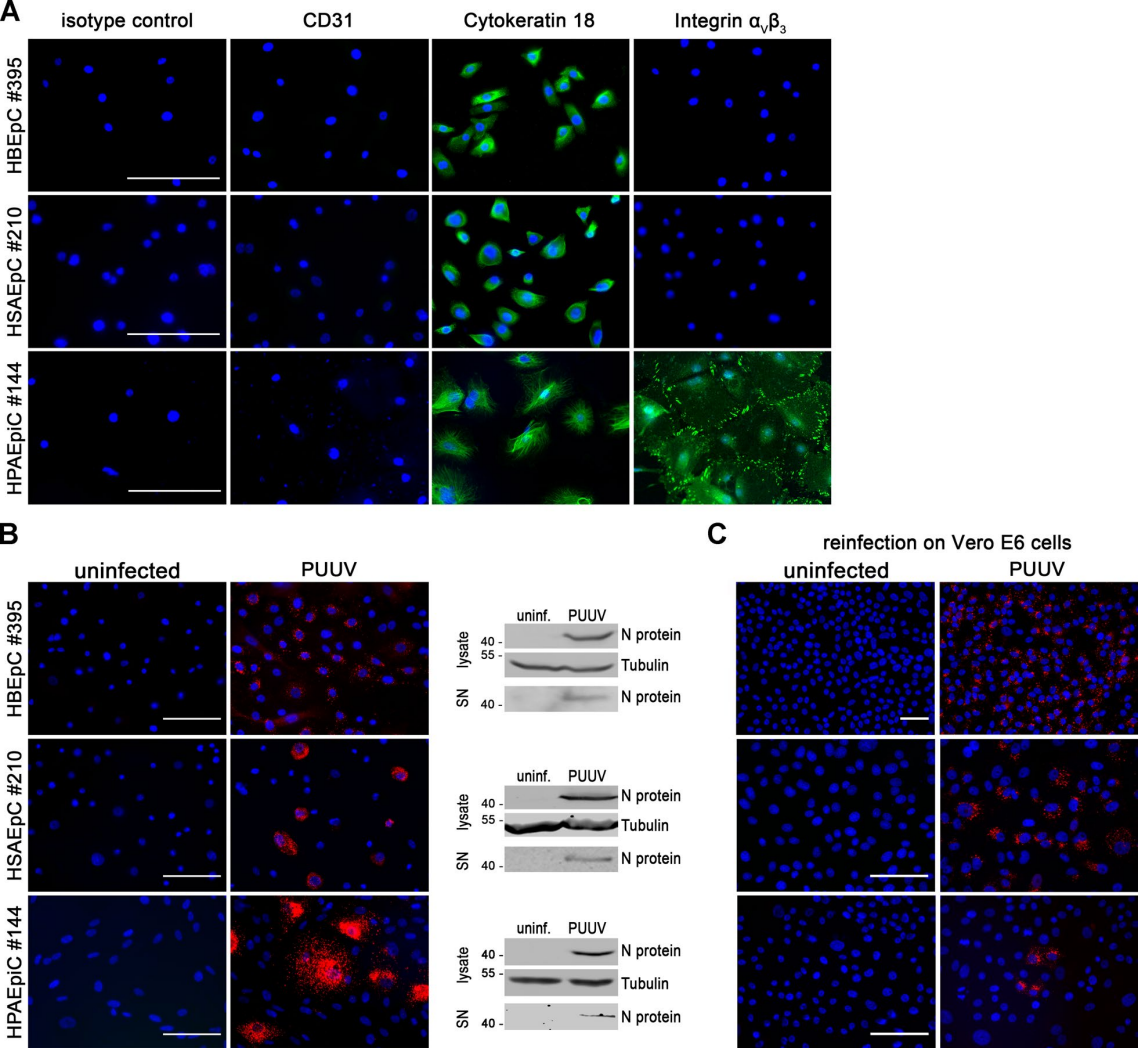
Infection of primary human pulmonary epithelial cells with PUUV. **A** Detection of marker proteins CD31, cytokeratin 18 and orthohantaviral receptor integrin α_V_β_3_ (scale bar: 100 µm). **B** HBEpCs, HSAEpCs, and HPAEpiCs were infected with PUUV. Infection and release were monitored by detection of N protein via immunofluorescence and via Western blot analysis of lysates and cell-free supernatants (SN) on day 6 post infection (scale bar: 100 µm). **C** Vero E6 cells were incubated with cell-free supernatants of uninfected or PUUV-infected pulmonary epithelial cells (6 dpi) and stained for N protein after 48 h post inoculation (scale bar: 100 µm)

**Fig. 3:**
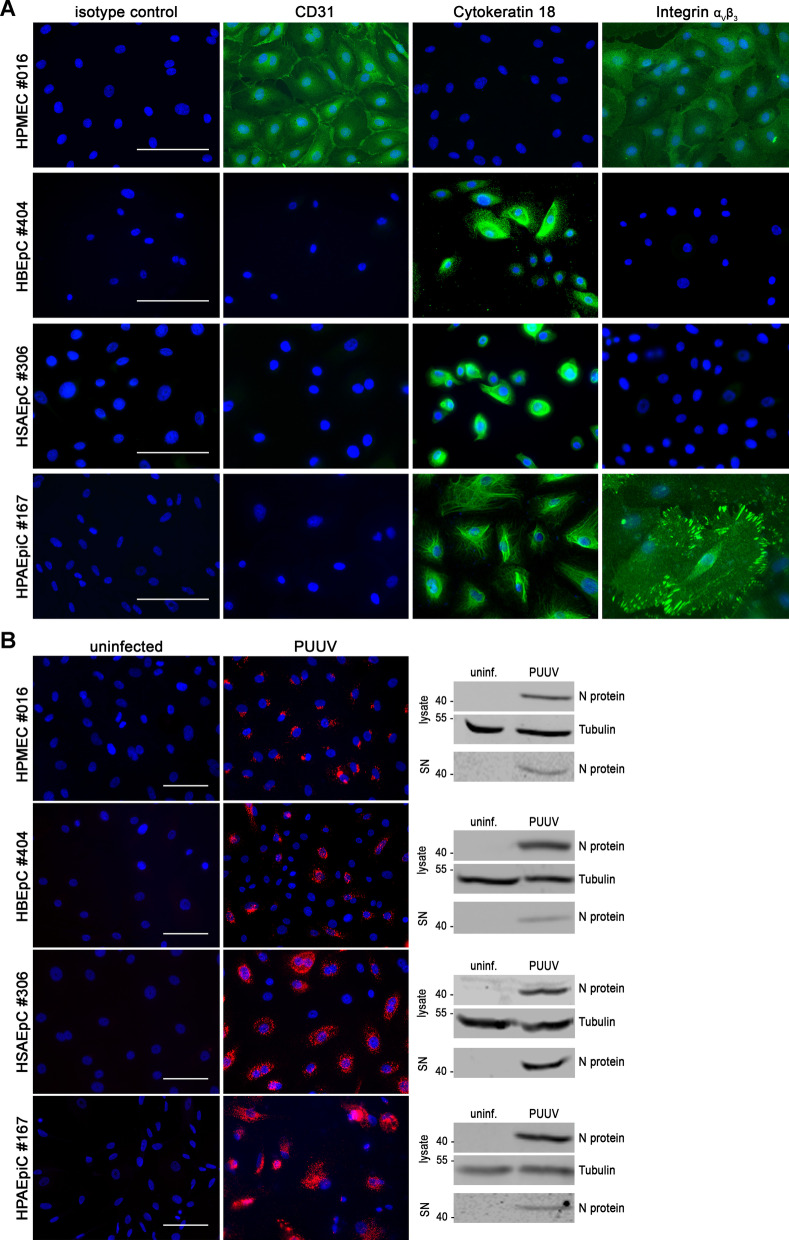
Analysis of human pulmonary cells from second donors for permissiveness to PUUV infection. **A** Detection of marker proteins CD31 and cytokeratin 18 and receptor integrin α_V_β_3_ by immunofluorescence (scale bar: 100 µm). **B** Pulmonary cells were inoculated with PUUV and analyzed for N protein expression on day 6 post infection. Cells were analyzed by immunofluorescence (scale bar: 100 µm). In lysates and cell-free supernatants (SN), N protein expression was detected by Western blot

Puumala virus (PUUV) strain Vranica isolated from bank vole [27, 28] was propagated and titrated on Vero E6 cells. For infection, virus inoculum (4.6 × 10^5^ IU/ml) was added at a multiplicity of infection (MOI) of 1 to primary pulmonary cells. After incubation for two hours at 37 °C, unbound virus was removed by triple washing with the appropriate medium. Infection was analyzed by immunofluorescence and Western blot analysis for orthohantaviral nucleocapsid protein (N protein). Infection kinetics were monitored by immunofluorescence staining of N protein and quantification of the percentage of positive cells. Experiments were performed in triplicates for each donor. Representative figures of day 6 post infection were shown in Figs. 1, 2 and 3.

### Quantification of viral release

To quantify the release of infectious particles from pulmonary cells, a first round of infection assay in Vero E6 cells was performed. Number of initially infected cells was quantified early after inoculation (48 h post inoculation) during the first replication cycle to avoid an overestimation of viral titers by second rounds of infection.

Cell-free supernatants from infected human pulmonary cells were collected on day two, four and six after infection and 10 µl of supernatant were added to Vero E6 cells for two hours. Inoculum was removed by a triple washing with medium. After 48 h, the total number of initially infected Vero E6 cells was quantified by immunofluorescence staining of PUUV N protein. The total number of infected Vero E6 cells corresponds to the number of infectious units (IU) in 10 µl. Titers were expressed as IU per ml supernatant. Experiments were performed in triplicates. Representative images of Vero E6 monolayers inoculated with supernatant of day 6 after infection were taken and shown in Figs. 1 and 2. All PUUV-related experiments were performed in a biosafety level 2 (BSL-2) setting.

### Immunofluorescence and Western blot

For immunofluorescence, cells grown on coverslips were fixed, permeabilized and stained with primary and appropriate fluorescently-labelled secondary antibodies. The following antibodies were used: mouse anti-CD31 (endothelial marker) (Dako), mouse anti-cytokeratin 18 (epithelial marker) (Millipore), mouse anti-PUUV nucleocapsid protein (Progen), and mouse anti-integrin α_V_β_3_, clone LM609 (orthohantaviral receptor) (Millipore). Cell nuclei were stained with Hoechst 33342 (Invitrogen). Images were taken using an Axiocam 506 camera attached to an Axio Observer.D1 microscope (Zeiss). For Western blot analysis, the following primary antibodies were used: rabbit anti-PUUV N protein, rabbit anti-integrin β_3_ (orthohantaviral receptor) (Santa Cruz) and mouse anti-α-tubulin (Sigma). Equal loading was verified by the detection of tubulin on the same membrane. Detection was performed by using near infrared fluorescent dye (IRDye)-conjugated secondary antibodies and an Odyssey infrared imaging system (Li-Cor Biosciences).

### Flow cytometry analysis of orthohantaviral entry receptors

Cells were washed, scraped and stained with allophycocyanin (APC)-conjugated mouse anti-CD55 (orthohantaviral receptor) antibody (BD Pharmingen) and phycoerythrin (PE)-conjugated mouse anti-integrin α_V_β_3_ (orthohantaviral receptor) antibody, clone LM609 (Millipore) or the corresponding isotype control. After one hour of incubation on ice, cells were washed and analyzed by flow cytometry with FACSCalibur (BD Pharmingen). Flow cytometry analysis of cell viability was monitored by using Via-Probe™ Cell Viability Solution (BD Pharmingen) according to manufacturer’s instructions.

### Statistical analysis

Data were analyzed using Prism 5.0 (GraphPad Software Inc.). Values of two groups were compared using two-tailed Student’s t-test. *P* values of < 0.05 were considered significant. *P < 0.05; **P < 0.005; ***P < 0.0005; ****P < 0.0001; ns: not significant.

## Results

### Infection of human primary pulmonary cells with PUUV

We examined human pulmonary microvascular endothelial cells (HPMEC) for their permissiveness and susceptibility to PUUV (Fig. 1). The endothelial identity of HPMEC was validated by the presence of the endothelial marker protein CD31 and the absence of the epithelial marker protein cytokeratin 18 (CK18). HPMECs were positive for CD31 and no expression of CK18 was detectable. Expression of orthohantaviral receptor integrin α_V_β_3_ was observed by immunofluorescence (Fig. 1A).

Inoculation of HPMECs with PUUV resulted in productive infection as shown by the detection of N protein in cells, lysate and supernatant (SN) (Fig. 1B). The release of infectious particles was demonstrated by reinfection of Vero E6 cells with cell-free supernatant derived from infected lung endothelial cells (Fig. 1C).

In a next step, we analyzed the infection of pulmonary epithelial cells of different parts of the respiratory tract, which represents a primary target epithelium for orthohantaviruses. HBEpCs isolated from human bronchi, HSAEpCs originating from 1 mm bronchiole area, and HPAEpiCs derived from alveoli were examined for marker proteins, receptor expression and permissiveness for PUUV infection (Fig. 2). Immunofluorescence analysis revealed that all epithelial cells were positive for the expression of the epithelial marker CK18 and negative for the endothelial marker CD31. In HBEpCs and HSAEpCs, expression of the integrin α_V_β_3_ heterodimer was not detectable. In contrast, expression of integrin α_V_β_3_ was present in HPAEpiCs (Fig. 2A).

Detection of PUUV N protein in cells and lysates six days after inoculation with PUUV demonstrated that all three pulmonary epithelial cell types were infected with PUUV (Fig. 2B). Infectious particles were released from pulmonary epithelial cells as shown by the detection of N protein in cell-free supernatants together with the reinfection of Vero E6 cells (Fig. 2B, C).

### Infection of pulmonary cells derived from second donors

To identify possible donor-specific effects in primary human pulmonary cells, cells of second donors were subjected to PUUV infection (Fig. 3). The endothelial and epithelial phenotype of all cell types was demonstrated by immunofluorescence staining for the endothelial marker CD31 and the epithelial marker CK18 (Fig. 3A). As observed for the first donors, all tested pulmonary cell types were permissive for infection with PUUV (Fig. 3B). The results of the receptor analysis in the second donors correspond to the observations made in the cells of the previous donors: expression of integrin α_V_β_3_ was not detectable in HBEpCs and in HSAEpCs and for HPMECs and HPAEpiCs, the expression of orthohantaviral receptor integrin α_V_β_3_ was confirmed in cells derived from second donors (Fig. 3A).

Together, our results demonstrate the permissiveness of human endothelial and epithelial pulmonary cells and the absence of detectable levels of integrin α_V_β_3_ in cells derived from bronchi and bronchioles.

### Receptor expression in human primary pulmonary cells

**Fig. 4:**
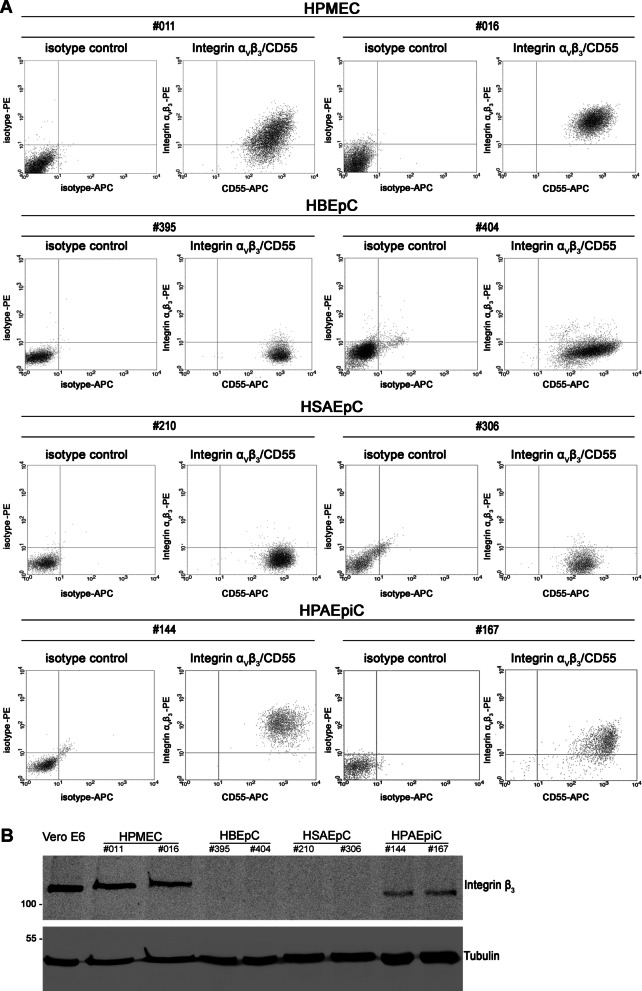
Receptor expression in human primary pulmonary cells from two donors. **A** Surface expression of integrin α_V_β_3_ and CD55 on human pulmonary cells was analyzed by flow cytometry. **B** Detection of integrin β3 subunit expression in lysates from human pulmonary cells by Western blot

To analyze the receptor expression in more detail, we examined surface expression of entry receptors by flow cytometric analysis (Fig. 4A). We stained pulmonary cells for integrin α_V_β_3_ and CD55, a co-receptor of orthohantaviral entry [15]. All pulmonary cells express CD55. Surface expression of integrin α_V_β_3_ was absent from HBEpCs and HSAEpCs and was present in HPMECs and HPAEpiCs. Integrins are heterodimeric receptors composed of α and β subunits and despite expression of β subunits, assembly and surface expression may be influenced by rate-limiting α subunits [29]. Therefore, we tested the expression of integrin β_3_ subunit in pulmonary cells by Western blot analysis (Fig. 4B). Integrin β_3_ subunit expression was not detectable in HBEpCs and HSAEpCs by Western blot. In contrast, integrin β_3_ subunit was observed in HPMECs and HPAEpiCs.

We showed the absence of detectable levels of integrin α_V_β_3_ surface expression as well as the lack of integrin β3 subunit expression in HBEpCs and HPAEpiCs. The results concerning absence and presence of receptor expression are consistent between donors.

### Replication kinetics of PUUV in human primary pulmonary cells

**Fig. 5:**
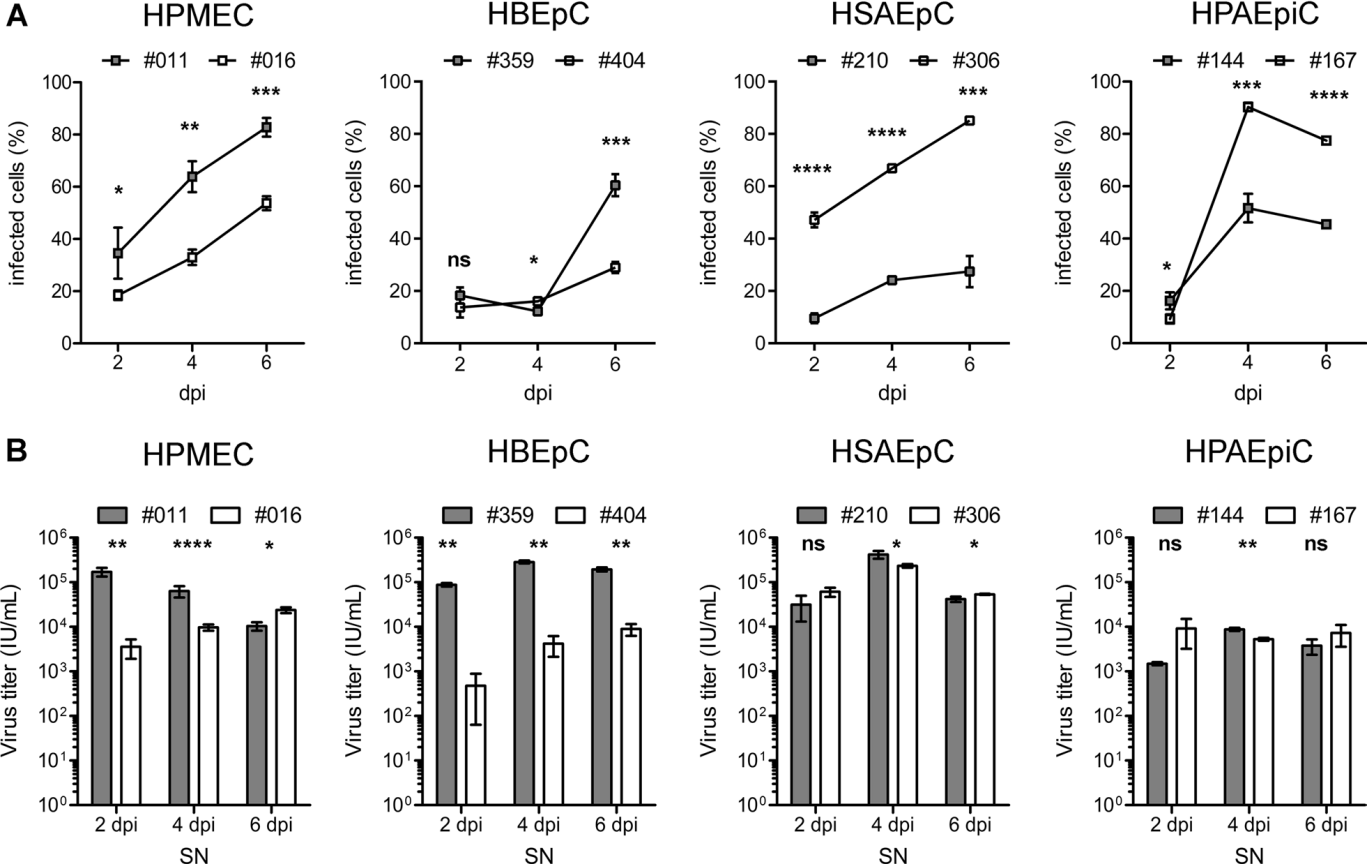
Replication kinetics of PUUV infection in human primary pulmonary cells. **A** Number of infected pulmonary cells was monitored by quantification of cells expressing N protein on day 2, 4 and 6 after inoculation. **B** Release of infectious particles was quantified via first round of infection assay by inoculation of Vero E6 cells with 10 µl cell-free supernatant (SN) derived from infected pulmonary cells on day 2, 4 and 6 after infection. Initially infected Vero E6 cells were quantified via immunofluorescence staining for N protein after 48 h post infection and the amount of infectious units (IU) per ml calculated. Experiments were performed in triplicates. Shown is mean ± standard deviation (SD). Differences between donors in mean percentage of infected cells and infectious units were compared using two-tailed Student’s t-test. *P* values of < 0.05 were considered significant. *P < 0.05; **P < 0.005; ***P < 0.0005; ****P < 0.0001; *ns* not significant

We directly compared kinetics of PUUV replication between different pulmonary cells types and between the two donors (Fig. 5 and Table 1). Number of infected cells and release of infectious particles were monitored over time by immunofluorescence-based assays. Western blot analysis of lysates and supernatants does not allow quantification due to possible cell type-specific differences between levels of N protein expression in infected cells and N protein detected in supernatants may be derived from defective non-infectious particles. The percentage of infected cells after initial infection increased in all cell types and donors and infectious particles were released over time. However, replication kinetics differ between pulmonary cell types. HPMECs, HSAEpCs, and HPAEpiCs demonstrated an increase of infected cells between day two and four after inoculation, whereas viral spread in HBEpCs started not before day four after infection. The percentage of infected cells reached its maximum at 6 dpi for HPMECs, HBEpCs, HSAEpCs of both donors. In contrast, the levels of infected HPAEpiCs peaked at 4 dpi and decreased at 6 dpi. The time course of replication kinetics is similar between donors of the same cell type, but donors demonstrate enormous variations in the percentage of infected cells. On day six post infection, 1.5- to threefold differences in the percentage of infected cells were observed between donors (Table 1). Analysis of titers of infectious particles revealed also significant donor-specific variation in viral release (Fig. 5B and Table 1). However, the amount of virion production is not related to the amount of infected pulmonary cells. Production of infectious particles may be reduced due to antiviral mechanisms of infected cells or by the production of defective non-infectious particles [30]. Nevertheless, number of infected cells may increase despite a decrease of viral release if infectious particles are present in excess compared to the number of permissive cells.

**Table 1:** PUUV infection and release of human primary pulmonary cells

Cell type	% infected cells (mean ± SD)			Infectious units/ml (mean ± SD)		
	2 dpi	4 dpi	6 dpi	SN 2 dpi	SN 4 dpi	SN 6 dpi
HPMEC #011	34.6 ± 9.8	63.9 ± 5.9	82.8 ± 3.6	1.7 × 10^5^ ± 3.8 × 10^4^	6.3 × 10^4^ ± 1.8 × 10^3^	1.0 × 10^4^ ± 2.2 × 10^3^
HPMEC #016	18.5 ± 1.8	33.0 ± 2.9	53.7 ± 2.7	0.4 × 10^4^ ± 0.1 × 10^4^	1.0 × 10^4^ ± 0.2 × 10^4^	2.4 × 10^4^ ± 0.4 × 10^4^
HBEpC #359	18.3 ± 3.1	12.2 ± 1.1	60.4 ± 4.2	8.8 × 10^4^ ± 8.0 × 10^3^	2.8 × 10^5^ ± 2.1 × 10^4^	1.9 × 10^5^ ± 2.1 × 10^4^
HBEpC #404	13.7 ± 3.8	16.0 ± 1.6	29.0 ± 2.1	4.8 × 10^2^ ± 4.1 × 10^2^	4.2 × 10^3^ ± 2.1 × 10^3^	8.9 × 10^3^ ± 2.7 × 10^3^
HSAEpC #210	9.6 ± 1.9	24.1 ± 1.1	27.4 ± 5.9	3.2 × 10^4^ ± 1.8 × 10^4^	4.2 × 10^5^ ± 8.2 × 10^4^	4.2 × 10^4^ ± 5.9 × 10^3^
HSAEpC #306	47.1 ± 2.8	66.8 ± 0.3	85.2 ± 0.4	6.1 × 10^4^ ± 1.4 × 10^4^	2.3 × 10^5^ ± 2.2 × 10^4^	5.4 × 10^4^ ± 1.6 × 10^3^
HPAEpiC #144	16.2 ± 3.2	51.7 ± 5.5	45.4 ± 1.3	1.5 × 10^3^ ± 1.2 × 10^2^	8.8 × 10^3^ ± 6.7 × 10^2^	3.8 × 10^3^ ± 1.4 × 10^3^
HPAEpiC #167	9.3 ± 1.8	90.3 ± 1.6	77.5 ± 0.4	9.1 × 10^3^ ± 5.9 × 10^3^	5.3 × 10^3^ ± 4.0 × 10^2^	7.3 × 10^3^ ± 3.7 × 10^3^

*SD* standard deviation, *SN* supernatant, *dpi* days post infection

In addition, there is apparently no relationship between the presence/absence of integrin expression and permissiveness of primary cells. Lowest initial infection rates were observed in HPAEpiCs #167 (9.3% ± 1.8%) that express integrin α_V_β_3_ and in HSAEpCs #210 (9.6% ± 1.9%) that have no detectable integrin α_V_β_3_ expression (Table 1). In addition, highest initial infection rates were also observed in cells with (HPMEC #011, 34.6 ± 9.8%) and without (HSAEpCs #306, 47.1% ± 2.8%) integrin α_V_β_3_ expression. Together, the permissiveness of pulmonary cells and donors varies enormously and does not depend on the expression of integrin α_V_β_3_.

## Discussion

The respiratory tract is the first region of contact for many pathogens. The ability of the pathogen to enter and to replicate in certain pulmonary cell types may influence the clinical picture and disease severity as shown for influenza virus and coronaviruses [31, 32]. However, the knowledge about initial target cells, replication sites and mode of dissemination to more distant target organs in human infection is often incomplete. By transmission via inhalation, pulmonary cells also represent possible target cells for human orthohantavirus infection. In chronically orthohantavirus-infected host rodents, lung tissue reveals the highest viral load [33]. Infected endothelial and epithelial cells of the human respiratory tract were detected in patients with New and Old World orthohantavirus infection [5, 34–36].

Cell culture infection studies in pulmonary cells also exist for orthohantaviruses. In vitro infection of human lung microvascular endothelial cells with SNV or HTNV in a 3D human lung tissue model results in the induction of chemokine expression and may contribute to an immune-mediated increase in vascular permeability [37]. Studies in cell culture models revealed the productive infection of hamster tracheal epithelial cells by New World orthohantavirus Andes virus (ANDV) without visible cellular damage [38]. The authors assume that replication in pulmonary cells may contribute to virus dissemination in the early steps of orthohantavirus infection. In vitro cell culture studies with Old World orthohantaviruses were often performed in the human pulmonary epithelial cell line A549 [39–42]. Interestingly, pathogenic orthohantaviruses replicate more efficiently in A549 cells than non-pathogenic species and infections with three subtypes of the species Dobrava-Belgrade orthohantavirus result in changes in the gene expression profile that correspond to the virulence of the respective virus subtype [39]. These results reflect the importance and suitability of human pulmonary epithelial cells as infection model for orthohantaviral pathogenesis. The infection of specific cell types in vitro is a useful tool to examine cell tropism, replication cycle and direct functional consequences, especially if relevant cells from human target organs were used [37, 43]. Of course, disease-specific effects may be caused by the cross-talk of cell types in a tissue-specific environment and by the activation of immune cells. These mechanisms cannot be properly analyzed in cell culture or tissue models. However, the detailed study of pathogenesis is still hampered by the lack of an adequate small animal model for Old World orthohantavirus infection [44]. Therefore, orthohantavirus research still depends on suitable human in vitro cell culture models to analyze replication cycle and pathogenesis.

Our work with human primary pulmonary endothelial and epithelial cells demonstrates the permissiveness of human cells within different parts of the respiratory tract for the Old World orthohantavirus PUUV. Epithelial and endothelial cells of the lung support orthohantaviral replication as observed for the infection of different cells from the kidney, which represent epithelial (tubular epithelial cells, podocytes) and endothelial (glomerular endothelial cells) cell types [7, 45, 46].

Interestingly, productive infection of bronchial and small airway epithelial cells occurred without detectable expression of integrin α_v_β_3_ or integrin β_3_ subunit. The absence of integrin β_3_ expression in normal human bronchial epithelium was also reported in several studies analyzing tissue samples or primary human bronchial epithelial cells [47–49]. Integrin β_3_ was described to serve as entry receptor for pathogenic orthohantaviruses by cell culture studies [13, 14]. In addition, there is a growing list of co-factors and receptors that have been identified in in vitro studies: CD55, gC1qR, protocadherin-1 or TIM-1 (T-cell immunoglobulin and mucin domain 1) were described to play a role in the entry of various orthohantavirus species in different cell culture models [15–17, 50–53]. Infection of several human cell types and of a PUUV host species cell line without integrin β_3_ expression was also observed [54–56]. Torriani et al*.* describe that orthohantaviral entry in human airway epithelial cells depends on macropinocytosis and identified differences between the New World orthohantavirus ANDV and the Old World orthohantavirus HTNV [57]. As a conclusion from these in vitro results, the entry of orthohantaviruses seems to be specific for different virus species and may also vary between cell types.

The replication analysis revealed differences in the kinetics of PUUV infection and release between donors. Donor-specific variation in permissiveness was also described for the infection of human airway epithelial cells with H1N1 Influenza virus and Nipah virus [58, 59]. The susceptibility and permissiveness for viral infection is influenced by host cell factors. Cellular antiviral response may account for differences in infection kinetics and may influence the clinical course and disease severity of viral infections. Differences in the individual immune response in patients infected with PUUV were observed [60, 61]. Behavioral factors (e.g. smoking) and genetic determinants such as HLA alleles or cytokine polymorphisms may contribute to the observed variation of immune response in infected individuals [62–67].

Based on the detection of orthohantaviral antigen in lungs of infected patients and the results from in vitro cell culture studies, pulmonary cells may represent target cells of orthohantavirus infection. The release of infectious particles indicates a possible viral spread in lung tissue. Nevertheless, a person-to-person transmission is not described for orthohantaviruses with the exception of ANDV [68–70]. The reasons for this remain unclear and were also discussed in the work of Pettersson et al., which describes the detection of PUUV RNA in the saliva of patients and a possible inhibition of virus transmission by salivary components [10].

In summary, the replication of orthohantaviruses in pulmonary epithelial cells may play a pivotal role in the initial phase of transmission, virus dissemination, and pathogenesis of pulmonary symptoms in orthohantavirus disease. Differences between donors and orthohantavirus species in pulmonary infection should be investigated in more detail in future work.

## Conclusions

Orthohantaviruses are transmitted via inhalation of contaminated aerosols and infections are characterized by specific manifestations in kidney and/or lung. As shown for renal cells, pulmonary cells may represent target cells of PUUV infection. The infection of bronchial and bronchiolar cells occurs in the absence of detectable integrin β3 expression indicating an alternate mode of entry in pulmonary cells. Patient-specific differences in the initial pulmonary replication as observed in vitro for cells from different donors may contribute to the severity of the clinical course. Therefore, the detailed analysis of orthohantaviral replication cycle in pulmonary cells is of special interest and will provide helpful insights in the mechanisms of orthohantaviral initial infection and its impact on pathogenesis.

## Data Availability

The datasets used and analyzed during the current study are available from the corresponding author on reasonable request.

## Abbreviations

ANDV: Andes virus
dpi: Days post infection
DOBV: Dobrava-Belgrade virus
HBEpC: Human bronchial epithelial cells
HCPS: Hantaviral cardiopulmonary syndrome
HFRS: Hemorrhagic fever with renal syndrome
HPAEpiC: Human pulmonary alveolar epithelial cells
HPMEC: Human pulmonary microvascular endothelial cells
HSAEpC: Human small airway epithelial cells
HTNV: Hantaan virus
IU: Infectious units
MOI: Multiplicity of infection
N protein: Nucleocapsid protein
PUUV: Puumala virus
SD: Standard deviation
SN: Supernatant
SNV: Sin Nombre virus
